# Depressive Symptoms and Deliberate Self-Harm in a Community Sample of Adolescents: A Prospective Study

**DOI:** 10.1155/2011/935871

**Published:** 2010-12-27

**Authors:** Lars-Gunnar Lundh, Margit Wångby-Lundh, My Paaske, Stina Ingesson, Jonas Bjärehed

**Affiliations:** Department of Psychology, Lund University, Box 213, 221 00 Lund, Sweden

## Abstract

The associations between depressive symptoms and deliberate self-harm were studied by means of a 2-wave longitudinal design in a community sample of 1052 young adolescents, with longitudinal data for 83.6% of the sample. Evidence was found for a bidirectional relationship in girls, with depressive symptoms being a risk factor for increased self-harm one year later and self-harm a risk factor for increased depressive symptoms. Cluster analysis of profiles of depressive symptoms led to the identification of two clusters with clear depressive profiles (one severe, the other mild/moderate) which were both characterized by an overrepresentation of girls and elevated levels of self-harm. Clusters with more circumscribed problems were also identified; of these, significantly increased levels of self-harm were found in a cluster characterized by negative self-image and in a cluster characterized by dysphoric relations to parents. It is suggested that self-harm serves more to regulate negative self-related feelings than sadness.

## 1. Introduction

Research shows that depression is relatively rare in children but becomes more prevalent in adolescence. At the same time, the sex ratio also changes considerably; whereas there is little evidence of gender differences in children, depression during adolescence is significantly more common among girls than boys [1, 2]. Another problem behavior that develops approximately at the same time is deliberate self-harm (henceforth referred to as self-harm), here defined as self-inflicted damage to the surface of one's own body. (It should be noted that the definition used in this study does not include behaviors like taking an overdose or self-poisoning. In this way, it is similar to the proposed diagnosis of “nonsuicidal self-injury” in the DSM-V [3], although our definition differs from this notion by not explicitly excluding suicidal intentions.). The mean age of onset of this kind of self-harm is reported to be around 12–15 years [4, 5], and self-harm is quite frequently reported among young adolescents [6–12]. This raises the question how depressive symptoms and self-harm are associated, both concurrently and prospectively. 

It is commonly assumed that self-harm develops as a symptom of high emotional distress (anxiety, depression, self-hate, etc.), as a way of expressing or regulating this distress [13–16]. This would mean that distressful emotions (e.g., depressive feelings) represent a risk factor for the development of self-harm. A risk factor is generally defined [17] as a measurable variable that must precede a negative outcome and be associated with a higher risk for this outcome, which means that risk factors can only be identified by means of prospective studies. So far, there are only a few prospective studies of emotional risk factors for self-harm. In one study [18], self-reported depressive symptoms at the age of 8 were found to predict acts of self-harm 10 years later in a community-based sample of 2,348 boys. In another study [19], predictors at the age of 12 for acts of self-harm at the age of 15 were studied; the results showed that self-reported internalizing problems and somatic complaints, as well as parent-reported externalizing problems and aggressiveness, independently predicted self-reported acts of self-harm 3 years later. 

In a recent study, however, we [20] failed to find support for emotional symptoms among 13–15-year-old adolescents as a risk factor for increased self-harm. In this study, emotional symptoms were measured by Goodman's [21] Strengths and Difficulties Questionnaire (SDQ). One problem with the SDQ, however, is that its 5-item subscale for measuring emotional symptoms contains only one depression-related item (whereas it contains three items related to nervousness, fear and worry, and one somatic item). It is likely that depressive feelings of guilt, shame, worthlessness, self-disgust, self-contempt, or self-hatred are more of a risk factor for the development of self-harm than are feelings of fear, worry, or nervousness. The SDQ emotional symptoms subscale may therefore be less optimal for detecting any existing prospective association between emotional problems and self-harm. It is quite possible that, although emotional symptoms in general do not serve as a risk factor for the development of self-harm, a *subset* of emotional symptoms do so. One purpose of the present study was to test the hypothesis that depressive symptoms represent such a subset of emotional symptoms.

Another purpose was to analyze subcategories of depressive symptoms and investigate if certain subsets of depressive symptoms, or patterns of such symptoms, are especially associated with self-harm. For example, because self-harm implies an attack towards one's own body, it may be assumed to be more associated with feelings of self-hatred, self-contempt, self-disgust, and so forth than with feelings of sadness, lack of energy, or difficulties in concentration.

Finally, we also wanted to test the possibility that self-harm may serve as a risk factor for increased depressive symptoms. In a previous study [20], we found evidence for a bidirectional relationship between self-harm and psychological difficulties in general; that is, overall psychological difficulties predicted an increase in self-harm one year later and self-harm predicted increased psychological difficulties one year later. Our hypothesis was that such a bidirectional relationship would apply also to the association over time between depressive symptoms and self-harm. There are several reasons to expect that self-harm may be a risk factor for increased depression. First, although self-harm may have an emotion-regulating function and therefore lead to decreased emotional distress as an immediate consequence [14], it may have the opposite effect in a longer time perspective by setting the stage for depression-related processes like rumination, shame, guilt, and regret. Second, when it comes to the attention of others (parents, friends, etc.) that an adolescent deliberately harms herself, these others may respond strongly negatively, thereby causing a deterioration and even disruption of interpersonal relationships in a way that may lead to depressive symptoms.

If there is a bidirectional prospective relationship between depression and self-harm, this would mean that depressive symptoms and self-harm may enter into a self-generating “vicious cycle” where increases in the one variable lead to increases in the other and vice versa. This can be described in terms of a dynamic system, where internal feedback processes lead to the emergence and stabilization of pathological patterns that include both depression and self-harm. Because evidence suggests that there may be gender differences in both depression and self-harm, we studied this question separately in girls and boys.

To summarize, the present study was carried out (1) to study if there is a bidirectional relationship between depressive symptoms and self-harm in young adolescents, in the sense that depressive symptoms serve as a risk factor for self-harm and vice versa; (2) to investigate different patterns of depressive symptoms, how frequent they are among young adolescents, and how they are related to self-harm. Methodologically, we combined two different approaches. First, we used a variable-oriented approach to study both concurrent and prospective associations between symptoms of depression and self-harm, with the hypothesis that we would find evidence of a bidirectional relation between depression and self-harm. Second, we used a person-oriented approach [22] in accordance with an advanced analytical procedure developed by Bergman [23] to identify different subgroups of adolescents with different patterns of depressive symptoms, and then compared these subgroups to see if they differed on self-harm. We hypothesized that the analysis would identify at least one clear depression-related cluster and that depression-related clusters would contain an over-representation of girls and be associated with significantly more self-harm than the other clusters. We also expected that adolescents characterized by some categories of depressive symptoms (e.g., negative self-related feelings) would show more self-harm than others (e.g., those characterized by sadness and lack of energy).

## 2. Materials and Methods

### 2.1. Participants

The participants were a community sample from a municipality in the south of Sweden which is fairly representative for the rest of Sweden in terms of the proportions of children living with both of their parents, and their ethnic backgrounds, but slightly more rural than Sweden as a whole, and with a slightly lower income level and educational level [24]. At Time 1, there were 532 students in Grade 7 (mean age 13.7 years) and 520 students in Grade 8 (mean age 14.7 years) in the schools of this municipality (excluding three special schools with place for around 25 individuals with severe school difficulties); 992 of these 1052 students (94%) participated. One year later, at Time 2, 984 students in Grade 8 and 9 participated. Ten individuals were excluded as multivariate outliers with stereotypic response patterns. In total, there were available longitudinal data for 879 participants (450 girls and 429 boys), who represented 83.6% of all students that were available for inclusion at Time 1.

### 2.2. Instruments

The participants filled out an 11-page questionnaire, which was tailor made for young adolescents and tested in a pilot study with around 200 participants [6, 24]. The questionnaire included a number of self-assessment instruments. The present study used data from four of these instruments, plus some separate questions. As the measure of deliberate self-harm, we used a short version of Gratz' [25] Deliberate Self-Harm Inventory. To measure depressive symptoms, a Depression Index was constructed on the basis of depression-related items from three separate instruments: the Strengths and Difficulties Questionnaire (SDQ [21]), a modified version of the Emotional Tone Index (ETI [26, 27]), and the Appearance subscale of the Body Esteem Scale for Adolescents and Adults, (BEAA [28]), with the addition of separate questionnaire items concerning sleep, alertness, self-rated health, and views on the personal future.

**Deliberate Self-Harm Inventory: 9-Item Version Revised (DSHI-9r)**

This is a shortened and modified version of the Deliberate Self-Harm Inventory which was originally constructed and validated by Gratz [25] and then translated into Swedish and adapted to adolescents [6, 8]. In this 9-item version of the DSHI, respondents are asked if they have deliberately engaged in any of nine different kinds of direct physical self-harm (cutting wrists, arms, or body areas; minor cutting causing bleeding; carving words, pictures, and so forth into the skin; burning oneself with cigarette, lighter, or match; severe scratching, causing bleeding; sticking sharp objects into the skin; biting oneself so that the skin is broken; punching oneself or banging one's head, thereby causing a bruise; preventing wounds from healing) during the past 6 months. Respondents are instructed to rate the number of times they have conducted these behaviours on a scale from 0 to 6, where 0 is “never” and 6 is defined as “more than five times”. A total score (from 0 to 54) on the DSHI-9r can thus be calculated by summarizing the number of times a person reports having engaged in these self-harming behaviours. The internal consistency of the DSHI-9r in the present study was *α* = .90. All nine items of the DSHI-9r correlated with mental health problems as measured by SDQ Total Difficulties (*r*s ranging from  .23  to  .33).

**Depression Index**

Depression-relevant items were selected from the 11-page questionnaire, according to their correspondence with items from standard measures of depression and the DSM-IV criteria for major depression [29]. Because the items came from different instruments with different response formats, the scores on each item were transformed to *z*-scores. Items referring to positive feelings were reverse scored. The items were then subjected to a principal components analysis with varimax rotation, which identified eight components with eigenvalues >1; on the basis of converging results from both a scree plot and parallel analysis, however, the number of components was reduced to 6. On the basis of these, six subscales were constructed (see the appendix): Dysphoric relations to parents (10 items of which 4 referred to positive feelings, alpha =  .85), Negative self-image (6 items of which 3 involved positive statements about the self, alpha =  .85), Dysphoric relations to friends (6 items which all referred to positive feelings, alpha =  .73), Fatigue/somatic complaints (5 items, alpha =  .70), Sadness/loneliness (4 items, alpha =  .67), and Difficulties in concentration (4 items, alpha =  .65). Test-retest correlations between Time 1 and Time 2 were *r* = .71 for the entire Depression index, *r* = .60 for Dysphoric relations to parents, *r* = .68 for Negative self-image, *r* = .51 for Dysphoric relations to friends, *r* = .61 for Fatigue/somatic complaints, *r* = .48 for Sadness/loneliness, and *r* = .61 for Difficulties in concentration.

### 2.3. Procedure

This research was conducted after approval by the Regional Ethics Committee at Lund University. Contact was established with school managements via head-masters who gave consent to their schools' participation in the study. Information about the form and purpose of the study was sent by mail to the parents, who were asked to contact the school teachers or the researchers if they did not want their child to participate. Parents as well as children were informed that this was a research project on the situation of young people today, in terms of how they feel, and how they perceive themselves, their feelings, relations, and life situation. The participants were also informed that their participation was voluntary, that they were free to withdraw at any time and without having to give a reason, that their answers were treated confidentially, and that no school personnel would have access to their answers. Contacts were established with representatives from school healthcare in the municipality to facilitate procedures if serious psychological problems or other circumstances related to participants would warrant an intervention. The procedure was considered ethically appropriate on the basis of previous research [30, 31].

The 11-page questionnaire was filled out in school, as part of a separate lecture hour, and was administered by research assistants from Lund University. A teacher was present, but did not participate in the data collection. In order to guarantee the students privacy, their school desks were separated as much as possible. The students were instructed to answer all questions as best they could, but not to think too much about any answer. They were instructed not to write their names anywhere on the questionnaire. After the completion of the questionnaire, it was sealed in an envelope by the student.

### 2.4. Statistical Analysis

The distribution of total DSHI-9r scores and the scores on two of the depression subscales (Dysphoric relations to parents and Sadness/loneliness) were highly positively skewed and leptokurtic at both Time 1 and Time 2; logarithmic transformations were therefore conducted on these three indexes, resulting in acceptable normal distributions. 

To test the hypothesis that depressive symptoms would be a risk factor for self-harm, we used both logistic regression (with incidence of new cases of repeated self-harm, at Time 2 as the dependent variable) and hierarchical linear regression (with DSH-9r scores at Time 2 as the dependent variable). To test the hypothesis that self-harm would be a risk factor for depressive symptoms at Time 2, we only used hierarchical linear regression. 

Cluster analysis was used to group all participants on the basis of their different profiles of scores on the six depression scales, according to the LICUR procedure [23]. This was done in four steps. First, multivariate outliers were identified by means of the residue procedure in the statistical package for pattern-oriented analyses SLEIPNER 2.1 [32] and removed from further analysis. Second, Ward's hierarchical clustering method was applied. Four criteria presented by Bergman [23] were used to decide on the optimal cluster solution: (a) theoretical meaningfulness of the cluster solution; (b) if there is a distinct drop in the explained error sum of squares (EESS) when a cluster solution is extracted, this suggests that two insufficiently similar clusters were merged to a nonoptimal cluster solution; (c) the number of clusters should not be more than 15 and should not be expected to be less than five; (d) the size of the EESS for the chosen cluster solution should preferably not be less than 67% and at the very least exceed 50%. In addition, the homogeneity coefficient of each cluster should preferably be <1. Third, a data simulation was undertaken to verify that the explained ESS was higher than what could be expected on a random data set with the same general properties as the data set used in the real analysis. Fourth, a nonhierarchical relocation procedure was carried out in order to improve the homogeneity of the clusters and to increase the variance explained by the cluster solution.

## 3. Results

As expected, girls showed more evidence of depression than boys; this did not, however, apply to all depression subscales. Gender comparisons by independent samples *t*-test showed that girls scored higher than boys on the total Depression index and on the subscales Negative self-image, Sadness/loneliness, and Fatigue/somatic complaints (all *P* < .001). On the other hand, the boys scored higher on Dysphoric relations to friends (*P* < .001), and there were no significant differences (*P* > .05) on Dysphoric relations to parents or on Difficulties in concentration. 

More girls than boys reported self-harm. At Time 1, 45.1% of the girls and 37.9% of the boys (*χ* ^2^ = 5.1,  *P* < .01) reported that they had harmed themselves deliberately at least once during the past 6 months. *Repeated* self-harm (defined as at least 5 instances of self-reported self-harm) was reported by 20.7% of the girls and by 15.9% of the boys. The stability of DSHI-9r scores from Time 1 to Time 2 was higher for girls (*r* = .57) than for boys (*r* = .35). 

Correlational analyses showed that total depression scores and self-harm were moderately to highly associated both in girls (*r* = .58 at Time 1 and *r* = .55 at Time 2) and boys (*r* = .39 at Time 1 and *r* = .46 at Time 2).

### 3.1. Prediction of New Cases of Repeated Self-Harm

Depressive symptoms at Time 1 predicted the incidence of new cases of repeated self-harm at Time 2 in both girls and boys. Incidence here refers to participants who reported repeated self-harm (defined as at least five instances of self-harm) at Time 2 but had reported *no instance of self-harm* at Time 1. The incidence rate of repeated self-harm was 10.4% (26 of 251) among the girls and 8% (21 of 254) among the boys. To test the hypothesis that depressive symptoms would predict the incidence of new cases of repeated self-harm, logistic regressions were carried out separately for girls and boys. As seen in Table 1, the model was significant in both genders (*χ* ^2^(1) = 9.13,  *P* = .003 in girls, and *χ* ^2^(1) = 5.56,  *P* = .018  in boys), explaining 7.3% of the variance in girls (*Nagelkerke R* ^2^ = 0.073) and 4.8% in boys (*Nagelkerke R* ^2^ = 0.048).

### 3.2. Bidirectional Associations between Self-Harm and Depression

The hypothesis of a bidirectional relationship over time between self-harm and depressive symptoms was confirmed in girls but not in boys. As seen in Table 2, hierarchical regression analyses among girls showed that, when controlling for self-harm at Time 1, depressive symptoms at Time 1 predicted self-harm at Time 2, and, conversely, when controlling for depressive symptoms at Time 1, self-harm at Time 1 predicted depressive symptoms at Time 2. In boys, however, there was only evidence for a unidirectional relationship; although depressive symptoms predicted self-harm, the reverse was not the case.

### 3.3. Cluster Analysis

In total, 977 participants had full data on the depression index at Time 1 and were entered into the cluster analysis. Of these, 24 adolescents were identified as multivariate outliers and excluded by the residue procedure. Second, the application of Ward's hierarchical clustering method, together with Bergman's [23] criteria, resulted in the choice of a ten-cluster solution, explaining 58.1% of the total error sum of squares (ESS). Third, a data simulation showed that the explained ESS of the cluster solution was significantly higher than expected by chance (*P* < .0001). Fourth, a non-hierarchical relocation procedure to improve the homogeneity of the clusters resulted in a ten-cluster solution that was found to explain 62.5% of the variance. The homogeneity coefficients of the clusters also were quite good, with most clusters having a homogeneity coefficient of <.40 and no cluster having a higher homogeneity coefficient than  .82 (low coefficients mean high homogeneity).

**Figure 1:**
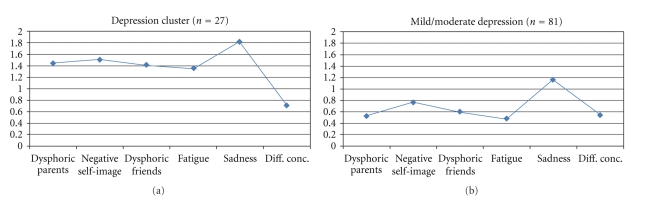
Depression-related clusters. Profiles in terms of *z*-scores (where *z* = 0 corresponds to the whole sample's mean on each subscale).

**Figure 2:**
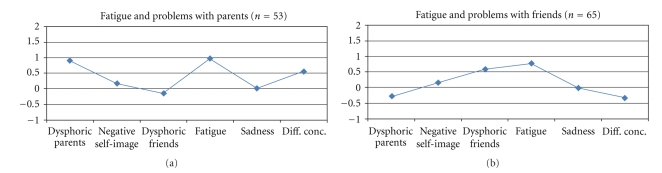
Two- and three-problem clusters. Profiles in terms of *z*-scores (where *z* = 0 corresponds to the whole sample's mean on each subscale).

**Figure 3:**
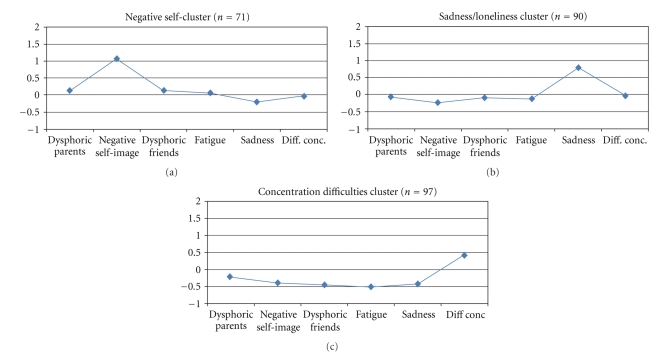
One-problem clusters. Profiles in terms of *z*-scores (where *z* = 0 corresponds to the whole sample's mean on each subscale).

**Figure 4:**
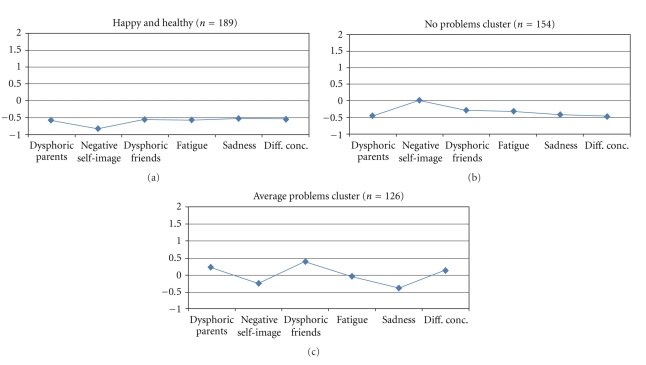
Healthy clusters. Profiles in terms of *z*-scores (where *z* = 0 corresponds to the whole sample's mean on each subscale).

Figures 1–4 show the profiles of *z*-scores for each cluster. As seen in Figure 1, the analysis identified two depression-related clusters: one small cluster (called the Depression cluster, *n* = 27), which was characterized by high scores on all depression indexes (*z* > 1 on all scales except on Difficulties in concentration), and one larger cluster (referred to as Mild/moderate depression, *n* = 81), which showed high scores (*z* > 1) on Sadness/loneliness and moderately high scores on all other subscales. As seen in Table 3, the Depression cluster showed a highly elevated depression score (*z* = 1.37), whereas the Mild/moderate depression cluster showed a moderately elevated score (*z* = 0.68).

In addition, there were a number of clusters that showed elevated scores on some depression indexes, without scoring high on total depression. As seen in Figure 2, there was a three-problem cluster that combined high scores on Fatigue/somatic problems, Dysphoric relations to parents, and Difficulties in concentration (called the “Fatigue and problems with parents” cluster, *n* = 53) and a two-problem cluster that combined high scores on Fatigue/somatic problems and Dysphoric relations to friends (called the “Fatigue and problems with friends” cluster, *n* = 65). As seen in Table 3, however, none of these clusters scored especially high on the total depression score.

As seen in Figure 3, three “one-problem clusters” were also identified: one with a highly negative self-image but normal scores on all other indexes (“Negative self-cluster,” *n* = 71), another with high scores only on Sadness/loneliness (“Sadness/loneliness cluster,” *n* = 90), and a third with high scores on Difficulties in concentration but relatively normal scores on the other indexes (“Concentration difficulties cluster,” *n* = 97).

Finally, the analysis identified three relatively large “healthy clusters” which together comprised 49.2% of the total sample: one cluster with low scores (*z* < −0.50) on all depression subscales (“Happy and healthy,” *n* = 189), another with relatively low scores on all indexes except Negative self-image where they scored close to average (the “No problems cluster,” *n* = 154), and a third with close to average scores on the indexes (the “Average problems cluster,” *n* = 126) (see Figure 4).

A one-way ANOVA with the ten-cluster categorization as the independent variable and the total depression index score as the dependent variable showed that the clusters differed on depression, *F*(9,943) = 641.1, *P* < .0001. Tukey post-hoc tests showed a categorization of the clusters into eight subsets, which differed significantly in the following order: Depression cluster > Mild/moderate depression cluster > “Fatigue and problems with parents” cluster > Negative self-cluster, “Fatigue and problems with friends” cluster > Sadness/loneliness cluster, and Average problems > Concentration difficulties cluster > No problems > Happy and healthy.

### 3.4. Gender Comparison between the Clusters

As expected, the girls were overrepresented in the depression-related clusters. The gender distribution in the ten clusters is shown in Table 3. To test the hypothesis that girls would be overrepresented in the depression-related clusters, the observed frequency was compared with the frequency that should be expected by chance alone, and one-tailed probabilities were computed according to the fixed-margins model using EXACON [33]. The results showed that the girls were overrepresented in both the Depression cluster (observed frequency 20, expected frequency 13.5, *χ* ^2^ = 3.13, *P* = .009) and in the Mild/moderate depression cluster (observed frequency 53, expected frequency 40.5, *χ* ^2^ = 3.86, *P* = .003). Explorative comparisons of the gender distributions in the eight remaining clusters, with the Bonferroni correction (*P* < .05/8 = .006), showed only one significant effect: boys were overrepresented in the Average problems cluster (observed frequency 89, expected frequency 63, *χ* ^2^ = 10.73, *P* < .0001, two tailed).

### 3.5. Comparison between the Clusters on Self-Harm

As expected, the depression-related clusters were associated with high levels of self-harm, but this was also the case for the “Fatigue and problems with parents” cluster and the Negative self-cluster. Table 3 shows the mean scores on the DSHI-9r for all the ten clusters. A one-way ANOVA with the ten-cluster categorization as the independent variable and the DSHI-9r as dependent variable showed that the clusters differed on self-harm, *F*(9,936) = 38.9,  *P* < .0001. Tukey post-hoc tests showed that the Depression cluster scored higher than all the other clusters. Further, the “Fatigue and problems with parents” cluster scored higher than six of the remaining clusters, the Mild/moderate depression cluster scored higher than five of the remaining clusters, and the Negative self-cluster scored higher than the three clusters with lowest DSHI-9r scores. 

To study the stability of these results, a similar one-way ANOVA was carried out with the DSHI-9r at Time 2 as the dependent variable. As seen in Table 3, the results were highly similar, showing that the Time 1 clusters differed significantly also on Time 2 self-harm, *F*(9,857) = 14.6,  *P* < .0001. Again, Tukey post-hoc tests showed that the Depression cluster scored higher than all the other clusters and that the “Fatigue and problems with parents” cluster scored higher than six of the remaining clusters. The Mild/moderate depression cluster and the Negative self-cluster also scored higher than the five clusters with the lowest self-reported frequencies of self-harm. 

## 4. Discussion

There are two main findings of the present study. First, there was support for the hypothesis of a bidirectional relationship between depressive symptoms and self-harm in the girls but not in the boys (where there was only support for a unidirectional relationship, depressive symptoms being a predictor of increased self-harm one year later). Second, and in line with expectations, among the ten profiles of depressive symptoms identified in the cluster analysis there were two depression-related clusters with an overrepresentation of girls and significantly increased levels of self-harm and also a single-problem cluster characterized by negative self-image and significantly higher levels of self-harm. 

Importantly, the demonstration of a bidirectional prospective relationship between depression and self-harm in girls means that higher levels of one of these variables *at a given time* are associated with increasing levels in the other variable *over time*. Whereas the concurrent analyses showed that *levels* of depression are strongly associated with *levels* of self-harm, the prospective analyses showed that higher levels of depression predispose to *increased* levels of self-harm within the next year and conversely that higher levels of self-harm predispose to *increased* depression within the next year. Although the effects were not strong, they are of a more dynamic order than the concurrent associations and suggest the possibility that depression and self-harm may enter into a self-generating “vicious cycle” where increases in the one variable lead to increases in the other, and vice versa. This can be described in terms of a dynamic system, where internal feedback processes lead to the emergence and stabilization of pathological patterns of depressive symptoms and self-harm. The absence of evidence for a bidirectional relationship in boys suggests that different developmental dynamics may be involved in girls and boys—or, in other words, that self-harm has a different meaning and function in girls than in boys.

In a previous study [20], we found no support for the hypothesis of emotional problems being a risk factor for the development of self-harm. In that study, emotional problems were measured by the Emotional symptoms subscale of the Strengths and Difficulties Questionnaire (SDQ). The present results corroborate the assumption that the SDQ Emotional symptoms scale is not sufficiently sensitive to capture the role of depressive symptoms for the development of self-harm, as it contains only one depression-related item.

There are several interesting results from the cluster analytic part of the present study. First, two depression-related clusters were identified, suggesting that 2-3% of the adolescents (27 of 953) in this community sample suffered from depression and that an additional 8-9% (81 of 953) suffered from something that is reminiscent of at least “minor depression.” Both of these clusters were characterized by an overrepresentation of girls (74% and 65%, resp.) and were also characterized by significantly higher frequencies of self-harm than most other clusters. The individuals in the depression cluster showed particularly high frequencies of self-harm. 

Second, the analysis identified three one-problem clusters of adolescents (the Negative self-cluster, the Sadness/loneliness cluster, and the Concentration difficulties cluster), characterized by high scores on one of the subscales without any elevated scores on the total depression index. In line with expectations, the Negative self-cluster was characterized by significantly increased levels of self-harm, whereas the other two were not. This is consistent with the hypothesis that self-harm is more associated with negative self-related emotions than with feelings of sadness. This finding also suggests the need for more specific theoretical models concerning the emotion-regulating role of self-harm. It is well established that a primary function of deliberate self-harm is the regulation of negative emotions [14]. But are all kinds of negative feelings (fear, sadness, shame, guilt, self-hate, etc.) equivalent in this respect, or are some emotional experiences more likely than others to be handled by self-harm? Because deliberate self-harm involves a direct physical attack on one's own body, and attack is more associated with anger or aggression than with fear or sadness, it may be hypothesized that self-harm is used primarily to regulate feelings of self-directed anger or aggression. It may be a task for future research to develop more specific theoretical models in this area and to develop instruments for testing these models. It should be noted that the items in the negative self-image subscale of the Depression Index in the present study were not constructed for this purpose and actually deal more with the absence of positive self-feelings than with the presence of negative self-feelings (see the appendix). The fact that this relatively weak index of a negative self-image was still able to produce results in line with the hypothesis suggests that this line of research may be worth pursuing.

A third finding from the cluster analysis is that high scores on fatigue/somatic complaints combined with dysphoric relations to parents and dysphoric relations to friends, respectively, into two separate problem clusters. For both of these, high scores on one of the dysphoric relations factors were combined with completely normal scores on the other. More specifically, the adolescents in the “Fatigue and problems with parents cluster” scored even slightly below average (*z* < 0) on dysphoric relations to friends. And, conversely, the adolescents in the “Fatigue and problems with friends cluster” scored slightly below average (*z* < 0) on dysphoric relations to parents. Whereas the “Fatigue and problems with parents” cluster had elevated scores on self-harm, this was not the case for the “Fatigue and problems with friends” cluster. This suggests that self-harm is more associated with negative relations to parents than with negative relations to friends. 

Fourth, the results from the cluster analysis gain further strength by their stability; the clusters defined at Time 1 showed highly similar results on self-harm also at Time 2. Still, it may be asked if the clusters with more circumscribed problems represent stable profiles or if the individuals in these clusters constitute risk groups for developing more depression later on. This could be studied by an analysis of the stability of these clusters from Time 1 to Time 2. 

A general comment is that the identification of subgroups like these probably cannot be done by variable-oriented approaches that rely only on the analysis of linear correlations; here, person-oriented methods like cluster analysis may represent an important complement, which make it possible to discover aspects of the data that are hard to detect otherwise. 

The present study has several strengths: it uses a large representative sample of adolescents, and there were longitudinal data for over 83% of all adolescents. The study, however, also suffers from several limitations. For example, the study used only two measure points, and it is possible that other results on risk factors would have been obtained if the first measure point had been earlier or if more measure points had been added. Another limitation is that the study relied entirely on self-assessment instruments; a multimethod approach might have made it possible to draw stronger conclusions. Further, the study did not use any established measures of depression but a specially constructed index based on items selected from different parts of a large questionnaire. A content analysis of the items (see the appendix) indicates that most of the criteria of major depression, as defined by the DSM-IV [29], are represented among the items. Two exceptions, however, are the DSM-IV criteria of weight loss or weight gain, and recurrent thoughts of death and suicide. This means that the depression index used in the present study does not do full justice to the psychiatric notion of major depression. On the other hand, the use of the present kind of depression index produced some interesting findings of potential interest to the understanding of adolescent depression, which would probably not have been seen if an established measure of depression had been used. For example, the present results identified dysphoric relations to parents and dysphoric relations to friends as two separate factors, with at least partly different meaning. One limitation with the dysphoric relations to parents subscale, however, is that all the items in this subscale refer to “parents” as the unit and do not allow for the possibility that some adolescents may feel differently towards their mother and father and hence be somewhat confused as to what to respond.

Finally, a possible risk with collecting data in school settings is that insufficient privacy may impact on levels of disclosure and thus threaten the validity and reliability of the results. An alternative possibility would have been to let the participants fill out the questionnaire at home; however, this would enter other ethical concerns and possible threats to response rates, as well as to validity and reliability. Also, the reports from the research assistants who administered the questionnaires gave no reason for concern about negative effects of lack of privacy but indicated that the students in general were very focused on the questionnaire during the lecture hour that was set off for filling it out. Further, the last page of the 11-page questionnaire included a question with a four-response format asking “how interesting and meaningful” the participants thought it had been to answer the questionnaire; the fact that over 80% of the students responded “very” or “fairly” to this question corroborates the impression that at least a large majority of the students were indeed engaged in the task.

## 5. Conclusion

To summarize, the present study contributes to the literature in at least two ways. First, it shows evidence of a bidirectional relationship over time between depressive symptoms and self-harm in young girls, although there was only evidence for a unidirectional relationship from depressive symptoms to self-harm among young boys. This suggests the hypothesis that depressive symptoms and self-harm in young girls may form a dynamic system, where feedback processes lead to the emergence and stabilization of self-generating “vicious cycles” of depressive symptoms and repeated self-harm.

Second, the present study uses cluster analysis in a way that gives a partly new perspective on aspects of adolescent depression and depression-related problem patterns. Apart from the identification of two depression-related clusters which were both characterized by an overrepresentation of girls and by elevated levels of self-harm, the analysis also identified a number of clusters with more circumscribed problems, and with different associations with self-harm. For example, the results also suggest that dysphoric relations to parents and to friends represent two separate dimensions that form part of different problem profiles and may be important to differentiate in order to understand the nature of depressive experiences in adolescence. Finally, the results indicate that negative self-image, sadness/loneliness, and difficulties in concentration exist as significant one-problem patterns in relatively large subgroups of adolescents, who do not show evidence of depression; of these, only the Negative self-cluster was associated with elevated levels of self-harm. The latter finding suggests that self-harm may serve to regulate negative self-related emotions rather than feelings of sadness; more research, however, is required on the kind of emotions that are associated with self-harm.

## Figures and Tables

**Table 1:** Logistic regressions, predicting incidence of repeated self-harm at Time 2 from depressive symptoms at Time 1, among participants with no self-harm at Time 1.

					95% Confidence intervals	
Variables	*B*	SE	Wald(1)	OR	Lower	Upper

*Girls*						
Depressive symptoms	.23	.08	9.20**	1.26	1.08	1.45
Constant	−2.09	.21	96.48***	.12		

*Boys*						
Depressive symptoms	.23	.09	5.85*	1.25	1.04	1.50
Constant	−2.30	.23	98.05***	.10

*P < .05, **P < .01, ***P < .001.

**Table 2:** Prospective hierarchical regressions, predicting T2 self-harm from T1 depressive symptoms and T2 depressive symptoms from T1 self-harm.

	Variables	*R* ^2^ * Δ*	*B *	SE B	*β*	*F step *
*Predicting self-harm at T2 from depressive symptoms at T1 among the girls *						

Step 1	T1 self-harm	.35	.61	.04	.60	247.1***
Step 2	Depressive symptoms at T1	.01				6.4*
	T1 self-harm		.55	.05	.53***	
	T1 depressive symptoms		.11	.04	.11*	

*Predicting depressive symptoms at T2 from self-harm at T1 among the girls *						

Step 1	T1 depressive symptoms	.54	.83	.04	.73	571.4***
Step 2	Self-harm at T1	.01				6.5*
	T1 depressive symptom		.77	.04	.68***	
	T1 self-harm		.12	.05	.10*	

*Predicting self-harm at T2 from depressive symptoms at T1 among the boys *						

Step 1	T1 self-harm	.17	.42	.04	.41	88.2***
Step 2	Depressive symptoms at T1	.03				52.8***
	T1 self-harm		.35	.05	.35***	
	T1 depressive symptoms		.17	.05	.18***	

*Predicting depressive symptoms at T2 from self-harm at T1 among the boys *						

Step 1	T1 depressive symptoms	.43	.79	.04	.65***	356.3***
Step 2	Self-harm at T1	.00				2.8
	T1 depressive symptoms		.76	.05	.63***	
	T1 self-harm		.08	.05	.06	

*P < .05, **P < .01, ***P < .001.

**Table 3:** Comparison between the clusters on gender, total depression score, and self-harm.

Cluster	*n*	Proportion	Total depr.	Self-harm	
		girls/boys	*z*-score	(DSHI-9r scores)	
Time	T1	T1	T1	T1	T2 (*n*)
Depression cluster	27	20/7	1.37	21.8	16.2 (21)
Mild/moderate depression	81	53/28	0.68	5.4	5.9 (73)
Fatigue and problems with parents	53	32/21	0.42	7.9	7.9 (43)
Fatigue and problems with friends	65	27/38	0.16	1.5	2.7 (56)
Negative self-cluster	71	45/26	0.20	4.8	5.5 (63)
Sadness/loneliness cluster	90	56/34	0.04	2.0	3.2 (84)
Concentration difficulties cluster	97	37/60	−0.08	2.3	2.5 (87)
Average problems cluster	126	37/89	0.01	2.6	2.2 (113)
No problems cluster	154	87/66	−0.32	0.8	1.9 (150)
Happy and healthy cluster	189	82/107	−0.58	0.8	1.0 (177)

## Appendix

### Items in the Depression Index and Its Subscales (with Response Format)

Dysphoric Relations to Parents
When I am with my parents (or think about them), I feel:

- sad, disappointed, depressed (Never/Seldom/Often/Very often)
- bored (Never/Seldom/Often/Very often)
- angry, irritated (Never/Seldom/Often/Very often)
- rejected, ignored, badly treated (Never/Seldom/Often/Very often)
- lonely, left out (Never/Seldom/Often/Very often)
- uneasy, restless (Never/Seldom/Often/Very often)
- calm, relaxed (Never/Seldom/Often/Very often) REV
- happy, joyous, glad (Never/Seldom/Often/Very often) REV
- a sense of belongingness (Never/Seldom/Often/Very often) REV
- liked, loved, cared for (Never/Seldom/Often/Very often) REV.

Negative Self-Image

- When I am with my parents (or think about them), I feel proud of myself (Never/Seldom/Often/Very often) REV
- I am ashamed of my looks (Never/Seldom/Often/Very often)
- I am rather satisfied with my appearance (Never/Seldom/Often/Very often) REV
- I wish I looked better (Never/Seldom/Often/Very often)
- I am proud of my body (Never/Seldom/Often/Very often) REV
- How do you think your life will be? (Very good/Rather good/Acceptable/Not very good/Not at all good).

Dysphoric Relations to Friends

- Other people my age generally like me (Not true/Somewhat true/Certainly true) REV.
- When I am with my closest friends (or think about them), I feel:
  - happy, joyous, glad (Never/Seldom/Often/Very often) REV
  - proud and sure of myself (Never/Seldom/Often/Very often) REV
  - a sense of belongingness (Never/Seldom/Often/Very often) REV
  - eager and full of energy (Never/Seldom/Often/Very often) REV
  - calm and relaxed (Never/Seldom/Often/Very often) REV.

Fatigue/Somatic Complaints

- Do you feel alert and energetic during the day? (Always/Most often/Sometimes/Seldom/Never)
- Do you sleep well? (Always/Most often/Sometimes/Seldom/Never)
- In general, how would you say your health is? (Very good/Fair/Poor)
- I get a lot of headaches, stomach aches, or sickness (Not true/Somewhat true/Certainly true)
- When I am with my parents (or think about them), I feel eager and full of energy (Never/Seldom/Often/Very often) REV.

Sadness/Loneliness

- I am often unhappy, down hearted, or tearful (Not true/Somewhat true/Certainly true).
- When I am with my closest friends (or think about them), I feel:
  - sad, disappointed, depressed (Never/Seldom/Often/Very often)
  - angry, irritated (Never/Seldom/Often/Very often)
  - lonely, left out (Never/Seldom/Often/Very often).

Difficulties in Concentration

- I am restless, I cannot stay still for long (Not true/Somewhat true/Certainly true)
- I am easily distracted, I find it difficult to concentrate (Not true/Somewhat true/Certainly true)
- I finish the work I am doing. My attention is good (Not true/Somewhat true/Certainly true) REV
- When I am with my closest friends (or think about them), I feel uneasy, restless (Never/Seldom/Often/Very often).
